# Development of a Japanese Sports Food Exchange List Reflecting Products Used in Japanese Athletic Settings

**DOI:** 10.3390/nu18111711

**Published:** 2026-05-27

**Authors:** Minami Isozaki, Moeka Nakamura, Yuya Kakutani

**Affiliations:** 1 Graduate School of Human Sciences, Osaka Shoin Women’s University, Higashi-Osaka 577-8550, Japan; 2 Department of Health and Nutrition, Osaka Shoin Women’s University, Higashi-Osaka 577-8550, Japan

**Keywords:** sports foods, athletes, food exchange list, energy, carbohydrates, protein

## Abstract

**Background**: Nutrient-enriched sports foods can support efficient nutrient intake in specific circumstances in athletic nutrition management, such as during competition, when training away from the usual environment, or during periods of weight management. Despite their widespread availability, sports foods are not always used appropriately, necessitating tools to support informed product selection. **Objective**: This study aimed to characterize sports foods consumed by Japanese athletes and to develop a Japanese sports food exchange list to facilitate product selection based on target nutrient requirements. **Methods**: Seven sports food categories commonly used in Japanese sports settings were examined: sports drinks, energy jellies, energy bars, energy gels, protein drinks, protein bars, and protein powders. Following the methodology of Spain’s sports food exchange list, development proceeded in two stages. First, suppliers were selected based on INFORMED CHOICE certification or listing on the Japan Anti-Doping Agency’s product information website, with input from experienced sports dietitians. Subsequently, 523 products were classified into subcategories based on nutrient content per unit using established statistical criteria, including the mean, standard deviation, coefficient of variation, and z-values. **Results**: After excluding products with z-values outside ±2 or compositions deemed unsuitable for carbohydrate or protein supplementation, 498 products from 36 suppliers were classified into 24 subcategories. Japanese sports foods exhibited broad distributions in nutrient composition, variability derived from ingredient differences, and a high proportion of plant-based protein powders. **Conclusions**: This study developed a Japanese sports food exchange list comprising 498 products across 24 subcategories, enabling evidence-based product selection aligned with the nutrient intake goals of Japanese athletes.

## 1. Introduction

In the field of nutritional strategies for athletes, the use of supplements and sports foods has been demonstrated to be an effective means of achieving adequate nutrient intake under specific circumstances. These include, but are not limited to, periods of competition, adherence to specific dietary patterns (e.g., veganism), and dietary restriction for weight reduction [1,2,3]. Sports foods are nutrient-enriched products, such as sports drinks and protein powders, that are intended for use during exercise [4]. In Japan, sports foods and dietary supplements are widely used by individuals ranging from elite international athletes to recreational exercisers [5,6]. Furthermore, studies of Japanese athletes have reported that 83.0% of Tokyo 2020 Olympic athletes and candidates and 78.7% of Beijing 2022 Winter Olympic athletes and candidates used sports foods [7]. These findings indicate that sports foods are extensively incorporated into athletes’ dietary practices.

Despite widespread use, supplements are frequently consumed inappropriately [8], and relatively few athletes select sports foods that align with their specific objectives [9,10]. Three primary challenges may underlie this issue. First, clear definitions and standards for sports foods are lacking. In Japan, regulations governing their definition, classification, and ingredient composition remain insufficient [5], resulting in substantial heterogeneity within product categories. For example, a previous study of sports drinks reported that the “sports drink” category on Japanese e-commerce platforms encompassed a wide range of products, including oral rehydration solutions and zero-calorie beverages [11]. Such ambiguity complicates the selection of products appropriate to specific nutritional needs. Second, packaging labels are complex. Label information and on-package claims strongly influence consumer choice [12], and the pervasive use of health and nutrition claims contributes to consumer confusion [13]. Third, athletes’ nutritional knowledge may be inadequate [14]. A systematic review of adult athletes identified misconceptions regarding both the necessity of supplements and the role of protein in skeletal muscle synthesis [15]. Similarly, a systematic review of youth athletes found that knowledge related to supplements was consistently the lowest among assessed domains, including food selection and hydration, across multiple studies [16]. These challenges are interrelated and collectively impede appropriate product selection. Accordingly, practical tools that enable athletes to select sports foods appropriately, irrespective of their level of nutritional knowledge, are needed to reduce unnecessary consumption and improve decision-making.

Food exchange lists have long been used as practical tools for dietary planning. These lists categorize foods into groups with approximately equivalent energy and macronutrient content, allowing substitution within groups. Since the introduction of exchange lists for diabetes patients in 1950, variants tailored to diverse applications have been developed worldwide, including for vegan diets [17], nutritional supplementation in malnourished populations [18], and baby food [19]. In Spain, a sports food exchange list has been developed for athletes [20]. However, this list reflects products available in the Spanish market only, limiting its applicability in other contexts. Our previous study [11] of the Japanese sports drink category identified 109 products, none of which overlapped with those included in the Spanish exchange list [20]. Furthermore, energy jelly, a product category widely available in Japan, is not included in the Spanish list [20]. For an exchange list to be practically useful in daily dietary planning, it must include products that are both readily available and commonly used in the target setting. Hence, the development of a context-specific exchange list reflecting sports foods available in Japan is warranted. This study aimed to identify sports foods used by Japanese athletes and to develop a Japanese sports food exchange list to facilitate product selection based on target nutrient requirements. This tool is intended to support more appropriate use of sports foods and thereby optimize performance.

## 2. Materials and Methods

This study was conducted in two stages: (1) selection of sports foods and domestic data collection and (2) classification of products into subcategories. This approach was based on the methodology used to develop Spain’s sports food exchange list [20,21,22].

(1) Definition of Sports Food

In this study, sports foods are defined as foods or beverages designed to provide athletes with convenient access to energy, carbohydrates, and protein within the context of nutritional support, particularly during exercise and in the pre- and post-exercise periods. This definition was established with reference to the consensus statements of the International Olympic Committee (IOC) [23], the supplement classification framework of the Australian Institute of Sport (AIS) [4], the Australia New Zealand Food Standards Code (FSANZ) Standard 2.9.4 [24], and the Japan Sport Association (JSPO) consensus on supplement use and application (2024) [25]. Although sports foods share overlapping characteristics with medical supplements and ergogenic aids, these categories differ in their primary purpose: medical supplements are intended for the prevention or treatment of clinically diagnosed nutritional deficiencies, while ergogenic aids are designed primarily to enhance performance. However, some products may span more than one category [26]. In this study, any product whose primary purpose is the supply of energy, carbohydrates, or protein was classified as a sports food, even if it also contained ingredients intended to enhance performance. The study focuses on sports foods widely used in Japanese sports settings [27]. Accordingly, seven categories were included: sports drinks (SD), energy jellies (EJ), energy bars (EB), and energy gels (EG) (carbohydrate-focused), and protein drinks (PD), protein bars (PB), and protein powders (PP) (protein-focused). Although sports confectionery and liquid meals fall within the broader definition of sports foods, both were excluded from this study. Regarding sports confectionery, conventional definitions proposed by the IOC and AIS refer to confectionery items specifically designed for sport; however, the range of products available in Japanese sports settings does not necessarily align with this definition, as it includes a diverse mix of products ranging from general carbohydrate-rich foods to protein-enriched snacks. Given this diversity, establishing a consistent definition of sports confectionery applicable to the Japanese context proved difficult, and these products were therefore excluded [4,23,24]. Liquid meals were also excluded, as the majority of products available in Japan are intended for medical or nursing care use.

(2) Selection and Data Collection of Domestic Sports Foods

1. Selection of Sports Food Suppliers

**Figure 1:**
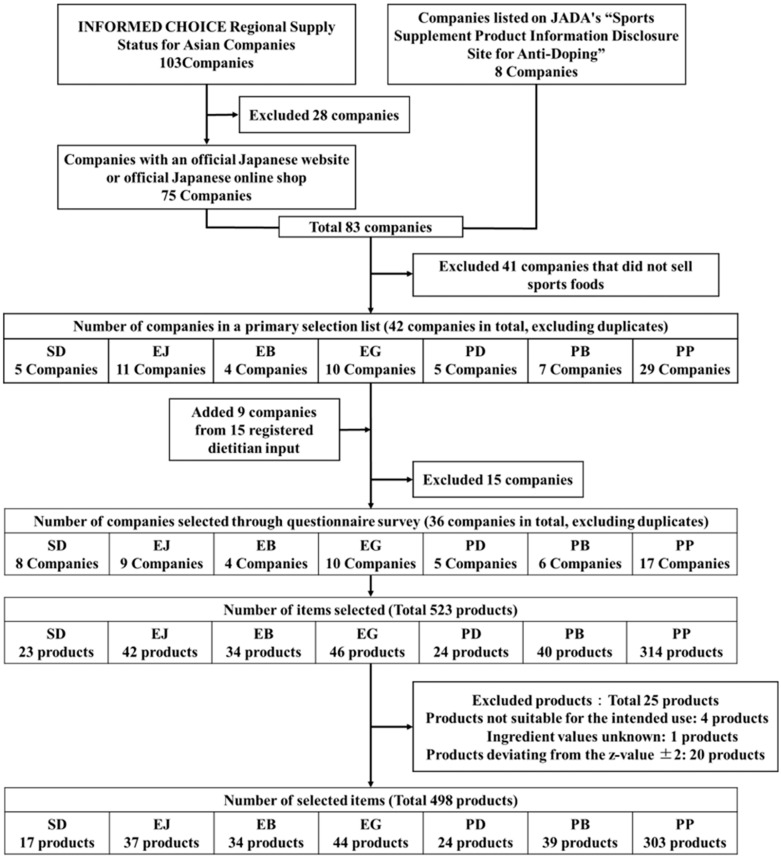
Selection of Target Products. SD: Sports drinks, EJ: Energy jellies, EB: Energy bars, EG: Energy gels, PD: Protein drinks, PB: Protein bars, PP: Protein powders.

To identify sports foods used in Japan, companies supplying these products were first identified (Figure 1). This study prioritized ensuring that athletes could safely use sports foods by focusing on products with low doping risk and meeting defined quality standards. Accordingly, companies holding Anti-Doping Certification [28] and those listed on the Japan Anti-Doping Agency (JADA) Product Information Disclosure Site [29] as of October 2024 were primarily selected. The procedure was as follows. From the INFORMED CHOICE certification list [28], operated by the UK-based Laboratory of Government Chemist (LGC), 103 companies with a regional supply status listed as Asia (as of 15 October 2024) were extracted. Of these, 75 companies whose official websites or official online stores were available in Japanese were targeted, as this criterion was applied to ensure that products were genuinely accessible to athletes in Japanese sports settings. Companies without their own sales sites and whose products were available only through third-party-operated online platforms were excluded because sufficient and verifiable nutritional information was often unavailable. Additionally, eight companies listed on JADA’s “Sports Supplement Product Information Disclosure Site for Anti-Doping” [29] that sold sports foods were included. This resulted in a primary selection list of 83 companies, all of which were extracted in October 2024. Of these, 42 companies were identified as selling sports foods and were subsequently included in the questionnaire survey administered to registered dietitians.

To reflect the common use of sports foods in competitive sports settings in Japan, input was obtained from 15 registered dietitians with experience supporting athletes in track and field (e.g., long-distance running), ball sports (e.g., soccer and basketball), weight-class sports (e.g., judo and boxing), and winter sports (e.g., skiing). Data were collected via an online Google Form, which presented the names of the 42 companies along with examples of their products and requested identification of companies supplying products commonly used in sports settings. Dietitians were also asked to provide free-text responses identifying additional companies not included in the primary selection list but whose products were commonly used by athletes, including company names and product details. This consultation involved no personal data and was conducted solely for product identification. The responses were integrated according to the following criteria: companies not included in the primary selection list were directly added to the final company list if two or more of the fifteen dietitians independently identified the same company or product. Companies mentioned by only one respondent, or frequently reported as unused by athletes, were referred to a panel of three experts in nutrition holding registered dietitian licenses for further deliberation. Through this process, companies were prioritized based on whether they supplied practical products that were actually used in Japanese sports settings and were readily accessible to athletes and dietitians, resulting in a final selection of 36 companies from which nutritional data were collected. The companies excluded from the preliminary selection list are shown in Table S1.

2. Selection of Sports Foods

Sports foods were then selected from the 36 target companies (Table 1) based on the following criteria: (1) classification into one of the seven sports food categories shown in Table 2; (2) clear labeling of energy, protein, fat, and carbohydrate content per product; (3) availability for purchase during the survey period from 27 March to 1 April 2025; and (4) suitability for room-temperature storage to withstand extended transport during travel or competition. Products with multiple flavors were treated as separate items, resulting in a total of 523 products.

3. Data Collection

For the 523 selected products, data were obtained from official company websites or product label images. Collected information included the supplying company, recommended single-serving size specified by the supplier, net content, and nutritional composition (energy, protein, fat, carbohydrates, and salt equivalent). Data collection was conducted between 27 March and 1 April 2025. Sports drinks and protein drinks were recorded in milliliters (mL), whereas other products were recorded in grams (g). For fat and salt values presented as ranges, median values were used. For fat content indicated as “less than XX,” the midpoint between 0 and the stated upper limit was used. For products lacking salt content (g) labeling, values were calculated by multiplying sodium (mg) by 2.54/1000. Nutritional composition data were standardized per unit, with one unit generally defined as one package. When the manufacturer specified a recommended serving size, this serving size was adopted as the unit. In this study, one unit served as the reference for comparing and organizing energy and macronutrient content, irrespective of product form or type. Unit definitions for each category, reflecting typical consumption by athletes, are presented in Table 2. In addition, INFORMED CHOICE certification status was verified using the LGC official website [28], and listing on JADA’s Sports Supplement Product Information Disclosure Site for Anti-Doping [29] was confirmed as of 1 August 2025.

(3) Classification into Subcategories

The seven categories comprising the 523 selected products were further subdivided into homogeneous subcategories based on macronutrient content. Classification followed the criteria for exchange list development proposed by Wheeler et al. [30], using distributions of energy and macronutrients per unit (mean, standard deviation, coefficient of variation, and z-scores). Products were grouped into subcategories with similar per-unit macronutrient values, enabling interchangeability (Table 3). During classification, efforts were made to assign products from the same brand to the same subcategory within the constraints of Wheeler et al.’s statistical criteria [30]. In addition to this approach [30], hierarchical cluster analysis using Ward’s method was conducted to explore objective classification. However, this method produced imbalanced groupings, including subcategories containing a single product, and occasionally assigned products from the same brand to different clusters. Because exchange lists are intended for practical use, such fragmentation reduces usability and complicates product selection. Consequently, only classifications based on Wheeler et al.’s criteria [30] were retained. Representative values for energy, protein, fat, and carbohydrate per unit were subsequently determined for each subcategory.

For the resulting 24 subcategories, cross-category exchanges for carbohydrate and protein supplementation were examined. Using the criteria proposed by Wheeler et al. (Table 3) [30], subcategory pairs were evaluated for interchangeability per unit within carbohydrate-supplementation and protein-supplementation categories, provided intake amounts were feasible. In addition to one-unit exchanges, exchanges involving one subcategory adjusted to two units were assessed. In such cases, the paired subcategory was fixed at one unit, and only combinations permitting interchangeability between one- and two-unit portions were considered.

(4) Development of a Booklet for Practical Application of the Exchange List

To promote the practical use of the developed Japanese Sports Food Exchange List, a booklet summarizing how to use the exchange list was created. The booklet was designed to enable athletes to select appropriate sports foods based on their competition level, activity level, and timing of intake, while also ensuring that key considerations for using the exchange list were easily understood by users. The content was finalized following a review and revision by two co-authors holding registered dietitian licenses.

(5) Usability Evaluation of the Sports Food Exchange Table

To evaluate the usability of the Japanese Sports Food Exchange Table we developed, we sought additional feedback from registered dietitians who had previously advised us on the product selection. The participants were asked to review the Japanese Sports Food Exchange Table and the accompanying booklet before providing their responses. The System Usability Scale (SUS) [31] was used for usability evaluation. The SUS consists of 10 items, each rated on a 5-point scale, with total scores ranging from 0 to 100. Responses were collected using a Google Form.

## 3. Results

We extracted nutritional composition data for 523 products from 36 target companies. To enhance within-category homogeneity, these products were classified into subcategories based on the statistical criteria of Wheeler et al. [30]. During this process, 20 products with z-values outside ±2, one product with missing nutritional data, and four products with compositions unsuitable for carbohydrate or protein supplementation were excluded. The final dataset comprised 498 products (Table 4, Table 5 and Table S2).

(1) Characteristics and Distribution of Nutritional Components in 498 Target Products

**Figure 2:**
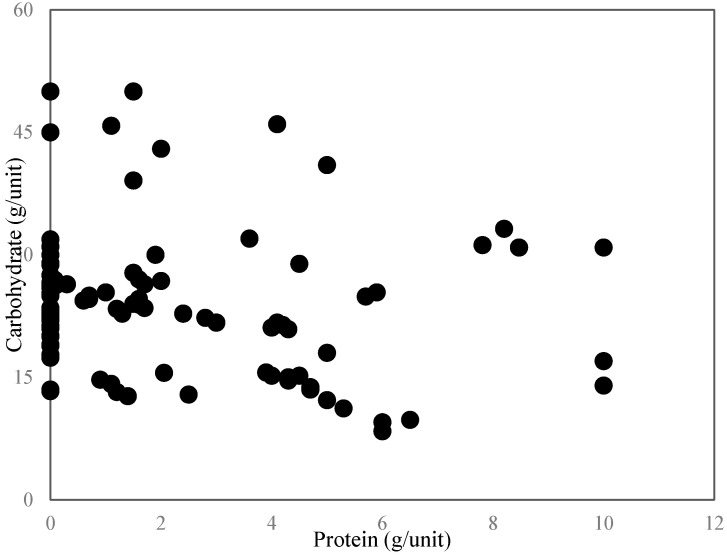
Average Protein and Carbohydrate Content per Unit of Sports Food for Carbohydrate Replenishment (*n* = 132).

**Figure 3:**
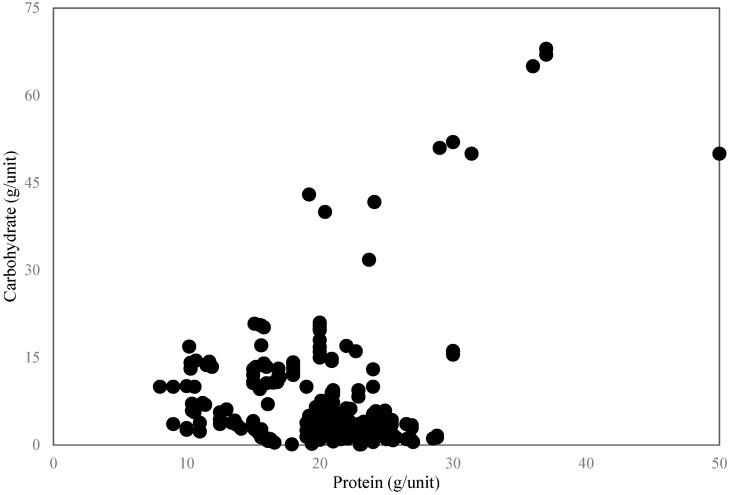
Average Protein and Carbohydrate Content per Serving of Sports Foods for Protein Supplementation (*n* = 366).

Figure 2 and Figure 3 show the distributions of protein and carbohydrate content per unit for the 498 products, focusing on those intended for carbohydrate and protein supplementation, respectively. The median carbohydrate content among carbohydrate-supplementing sports foods was 23.2 g/unit, with products containing 15–30 g/unit accounting for 69% (91 products). Among these products, 50% (66 products) contained 0 g of protein per unit. However, within the energy gel category, five products contained 8–10 g of protein per unit. The median protein content among protein-supplementing sports foods was 20.2 g/unit, with products containing 10–25 g/unit accounting for 93% (339 products). Although the median carbohydrate content was 9.8 g/unit among protein-supplementing sports foods, in protein powders with “gainer” in their product names, eight protein powder products labeled as “gainer” contained more than 50 g/unit of carbohydrates.

(2) Nutritional Composition Characteristics of the 24 Subcategories

Table 6 presents the per-unit nutritional composition of the 24 subcategories derived from the 498 products. Each subcategory comprises products with similar nutritional profiles, allowing interchangeability within subcategories. In the carbohydrate supplementation categories, representative protein and fat values were 0 g/unit in Sports Drink Subcategory 2 (SD2) and Energy Gel Subcategories 1 and 2 (EG1 and EG2).

The representative nutritional values for carbohydrate-supplementing sports foods are shown in Table 6. Sports drinks showed limited variation and were thus classified into two subcategories. Subcategory 1 (SD1) included products containing protein, whereas Subcategory 2 (SD2) contained higher carbohydrate levels per unit than SD1. Energy jellies were classified into three subcategories: Subcategory 1 (EJ1) had the lowest energy (98 kcal/unit) and carbohydrate content (24 g/unit); Subcategory 2 (EJ2) had the highest carbohydrate content (46 g/unit); and Subcategory 3 (EJ3) contained 8.7 g/unit of protein and 3.5 g/unit of fat. Energy bars were also divided into three subcategories: Subcategory 1 (EB1) had the lowest energy (98 kcal/unit) and carbohydrate content (16 g/unit); Subcategory 2 (EB2) had higher carbohydrate content (23 g/unit) and higher fat content (10 g/unit) than EB1; and Subcategory 3 (EB3) comprised three products with low fat content. Energy gels were classified into three subcategories: Subcategories 1 (EG1) and 2 (EG2) contained no protein or fat and differed in carbohydrate content, whereas Subcategory 3 (EG3) contained both protein and fat.

**Figure 4:**
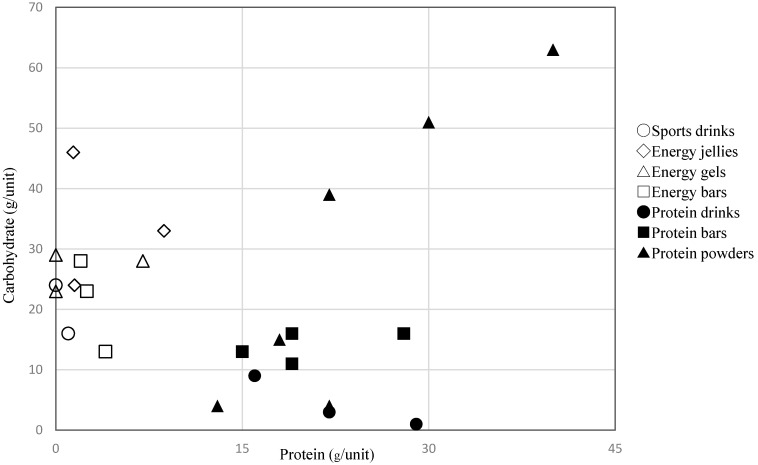
Protein and Carbohydrate Content per Exchange Unit for All 24 Subcategories of the Japanese Sports Food Exchange List (*n* = 24).

The characteristics of subcategories for protein-supplementing sports foods are also shown in Table 6. Protein drinks were classified into three subcategories: Subcategory 1 (PD1) had the highest carbohydrate content per unit, while Subcategories 2 (PD2) and 3 (PD3) had higher energy and protein content than PD1, with only PD2 containing fat. Protein bars were classified into four subcategories: Subcategory 1 (PB1) had the lowest energy and protein content; Subcategories 2 (PB2) and 3 (PB3) shared a representative protein value of 19 g/unit, with PB2 having higher carbohydrate content and PB3 having higher fat content. Protein powders were subdivided into six subcategories to improve homogeneity, reflecting substantial variation in composition. Subcategory 1 (PP1) had the lowest protein content (13 g/unit) among protein-supplementing sports foods. Of the 303 protein powder products, 44 were plant-based, primarily derived from soy or peas, and 64% (28 products) of these were classified into PP1. Subcategories 2 (PP2) and 3 (PP3) had protein contents of 18 g/unit and 22 g/unit, respectively, with PP2 showing higher carbohydrate content (15 g/unit) than PP3. Subcategory 3 (PP3) encompassed 224 products, representing the largest group. Subcategory 4 (PP4) had the highest carbohydrate and protein content per unit among all subcategories. Subcategories 5 (PP5) and 6 (PP6) also exhibited high carbohydrate and protein content, although PP6 had a smaller unit quantity than PP4 and PP5. Among products classified into PP4, PP5, and PP6, those labeled “weight gainer” or “lean gainer” accounted for 91% (11 of 12 products). Figure 4 illustrates the distribution of protein and carbohydrate content per unit across all 24 subcategories, highlighting differences between carbohydrate- and protein-supplementing sports foods. In protein-supplementing categories, carbohydrate content ranged from 1 to 63 g/unit, indicating substantial variability. Conversely, in carbohydrate-supplementing categories, protein content ranged from 0 to 8.7 g/unit. Table 7 presents exchange pairs across categories based on supplementation purpose. Within protein-supplementation subcategories, large differences in energy and carbohydrate content limited interchangeability, resulting in only one exchangeable pair.

(3) Usability Evaluation of the Sports Food Exchange Table

The SUS scores for the Sports Food Exchange Table are presented in Table 8. Responses were received from six registered dietitians, and the average SUS score was 53 points.

In the open-ended comments, respondents noted that “for dietitians and others with nutritional knowledge, this is a tool that makes it easy to organize the selection of sports foods according to specific goals,” suggesting that this exchange table has the potential to be a useful tool for supporting product selection in sports nutrition practice. On the other hand, some comments indicated that “when athletes use it themselves, they need to have a certain level of understanding of basic nutritional knowledge, such as carbohydrates and proteins,” suggesting a need for support tailored to the user’s level of knowledge.

## 4. Discussion

This study aimed to develop a Japanese version of the Sports Food Exchange List to support dietary planning by reflecting sports foods used in Japan.

The exchange list developed in this study indicates that sports foods used in Japan are characterized by a wide distribution of nutritional composition. In particular, among sports foods intended for protein supplementation, protein and carbohydrate content varied substantially by category. In Spain’s sports food exchange list [20], the protein powder category comprised two subcategories based on protein content (23 g/unit and 26 g/unit). In contrast, the present study classified products into six subcategories ranging from 13 g/unit to 40 g/unit. Furthermore, protein powders classified as PP4, PP5, and PP6 were characterized by larger unit masses and high carbohydrate and protein contents per unit. Additionally, across sports foods intended for protein supplementation, approximately 20% of products contained more than 10 g of carbohydrate per unit, indicating that many products are designed to provide both protein and carbohydrate simultaneously (Figure 3). Such products may be advantageous during post-exercise recovery, when rapid glycogen resynthesis and stimulation of muscle protein synthesis are both desirable. However, their nutritional significance is context-dependent. Even within the same product, its role may differ depending on whether it is used as a meal replacement or as part of a strategy to promote body mass gain. Accordingly, classifications based solely on nutritional composition should be interpreted alongside practical considerations such as the timing of intake and body weight management objectives. This compositional diversity suggests a wide range of applications and target users for sports foods in Japan and indicates increased complexity in product selection for dietary planning.

A second characteristic is variation in nutritional composition attributable to differences in ingredients. In bar-shaped sports foods, both the Spanish study [20] and the present study observed higher fat content compared with other product forms. This reflects the use of ingredients such as peanut butter and nuts, which contribute fat not only to enhance palatability and satiety but also to provide energy efficiently [32,33]. However, in the Japanese exchange list, EB3 includes the energy bar “Enemochi,” which uses mochi (rice cake) as its primary ingredient and exhibits lower fat content than other bar products. Thus, even within bar-shaped sports foods, nutritional composition varies considerably depending on ingredient composition. Similarly, products containing okara (soy pulp) were included among the target products. The presence of sports foods incorporating ingredients characteristic of Japanese cuisine, such as mochi and okara, suggests that national food culture influences sports food development. Another notable feature is the relatively high proportion of plant-based protein powders, primarily derived from soy or peas. In Spain’s sports food exchange list [20], plant-based protein powders accounted for 7% (five products) of the total protein powder category, whereas they accounted for 15% (44 products) in the present study. Furthermore, a study of the protein powder market in Chile reported that plant-based products accounted for only 1% [34]. These comparisons indicate that the proportion of plant-based protein powders in Japan is substantially higher than in other countries. This pattern may reflect Japan’s traditional dietary reliance on soy-based foods, as well as increasing health and environmental awareness [35,36]. In the present study, 64% (28 products) of the 44 plant-based protein powders were classified into PP1, with a protein content of 13 g/unit, which is lower than that of other subcategories. PP1 represents the lowest protein content among sports foods intended for protein supplementation, indicating that plant-based protein powders were predominantly classified into this lower-protein subcategory. Although these international comparisons should be interpreted with caution, as differences in market environments, product classification approaches, and cultural dietary practices, sports foods used in Japan exhibit both compositional diversity and ingredient-specific characteristics. These results imply the need for a Japan-specific exchange list distinct from existing international models.

Moreover, the Japanese sports food exchange list may better reflect the practical sports environment in Japan than the Spanish sports food exchange list [20]. The present list includes 498 products, compared with 322 in the Spanish list [20], thereby offering a broader range of options. While the Spanish list includes sports confectionery and liquid meals, these products are less common in the Japanese market. To enhance practical applicability, the present study focused on commonly used products and included energy jellies in place of sports confectionery and liquid meals. Energy jellies were classified into three subcategories with representative carbohydrate values of 24 g/unit (EJ1), 46 g/unit (EJ2), and 33 g/unit (EJ3). These values are comparable to or higher than those of other carbohydrate-supplementing sports foods, with EJ2 providing the highest carbohydrate content (46 g/unit), indicating its suitability for carbohydrate supplementation.

Considering these characteristics, the exchange list was developed with an emphasis on simplicity and usability in sports settings. In this study, products were first classified into seven categories, and then further divided into subcategories based on the nutritional composition of each product. This approach is expected to minimize the variability in nutritional composition within subcategories, thereby reducing the risk of inappropriate intake or errors in product selection. During subcategory classification, nutritional composition was prioritized as the primary basis for classification, and efforts were made to assign products from the same brand to the same subcategory within the allowable range established by Wheeler et al. [30]. This approach was intended to improve convenience for athletes and dietitians when exchanging products within the same brand, and to clarify brand-specific nutritional composition and ingredient characteristics, thereby facilitating product selection. It should be noted that some products were classified without strict statistical methods, reflecting the emphasis placed on practical usability. Nevertheless, all products were classified to meet the criteria defined in Table 3 for their respective target nutrients (carbohydrates or protein), ensuring a balance between usability and nutritional validity. Although the Japanese Sports Food Exchange List primarily enables exchanges within subcategories, exchanges across categories are possible for certain subcategories. However, such cross-category exchanges were limited in number, particularly among sports foods intended for protein supplementation, reflecting the substantial variability in nutritional composition across products. Therefore, this exchange list should primarily be interpreted as a tool for product selection and exchange within subcategories rather than as a broadly interchangeable system across categories. However, several considerations are required when using the exchange list. First, regular foods should be prioritized, and overall meal planning should be established before incorporating sports foods. This exchange list was developed for athletes at competitive levels and activity volumes at which sports foods may be integrated into dietary planning. Specifically, the intended population corresponds to Tier 2 or higher athletes, as defined by McKay et al., representing regional-level athletes who train regularly approximately three times per week [37]. In addition, when using sports foods, it is necessary to evaluate whether the benefits outweigh the potential risks of anti-doping violations [23]. Appropriate exchanges must be performed based on target nutrient requirements and current intake status. Particular caution is required when exchanging sports drinks. During prolonged exercise in hot environments, the intake of fluids and electrolytes, in addition to carbohydrates, is recommended [38]. In this study, sports drinks had a higher mean salt equivalent content (0.6 g/unit) than other sports foods, reflecting their role in electrolyte replacement. Therefore, substituting sports drinks with products intended solely for carbohydrate supplementation may hinder adequate fluid and electrolyte intake. Attention is also required regarding variation in serving size per unit across products. For example, protein powder serving sizes ranged from 16 g/unit (PP1) to 133 g/unit (PP4), necessitating confirmation of feasibility for actual consumption. Accordingly, differences in protein content across subcategories have important practical implications. PP1, which primarily contains plant-based protein, and PP2, which is intended for weight management, provide relatively low amounts of protein per unit and may not meet the recommended 20–40 g per meal for maximizing muscle protein synthesis [39]. Although PP3 falls within this range on average, it may be insufficient in a single unit for larger athletes, potentially requiring multiple units depending on body size and nutritional goals. Therefore, appropriate use of the exchange list requires a clear understanding of both product characteristics and the intended nutritional purpose. Furthermore, the sports foods examined in this study were limited to products available between 27 March and 1 April 2025; therefore, product availability and nutritional composition may have changed. Verification of current product information at the time of use is recommended.

The Japanese Sports Food Exchange Table developed in this study is directly linked to key nutritional strategies in sports nutrition and serves as a tool to support decision-making in practical settings. Sports foods are particularly effective in certain situations regarding carbohydrate replenishment strategies. While daily carbohydrate intake is generally supplied through regular foods and meals, during or immediately after exercise, regular foods are not always the most appropriate choice from the perspectives of portability, digestion and absorption. In such situations, the use of sports foods is effective, and this exchange table systematically supports the selection of appropriate sports foods. Carbohydrate intake during exercise depends on the exercise duration; for prolonged exercise lasting 1 to 2.5 h, 30 to 60 g/h is recommended [40]. For example, assuming a carbohydrate intake of 45 g/h, using this exchange table allows for the selection of products appropriate to the situation from multiple options such as consuming 1 unit of EJ2, or a combination of 1 unit of SD1 and 1 unit of EG3, demonstrating that the exchange table serves as a practical guide for product selection in the field. This exchange table also plays a useful role in protein intake strategies. Even if the total daily protein intake is sufficient, an uneven intake across meals may hinder the maximization of muscle protein synthesis. Consuming 20–40 g of protein per meal is considered effective for muscle protein synthesis [41], and the protein supplementation subcategories in this exchange table allow for product selection based on this target amount. In particular, in situations where protein intake tends to be uneven due to a busy training schedule, referring to the representative protein values for each subcategory allows for the rapid selection of products to compensate for any deficiencies.

This study has several limitations. First, nutritional composition was not directly analyzed but derived from label information provided by suppliers; therefore, discrepancies between labeled and actual values cannot be excluded. Additionally, the use of midpoint values for products labeled with ranges or “less than” values may introduce minor estimation error. However, these products typically contained low nutrient amounts, and the use of midpoint values was therefore unlikely to have materially affected subcategory classification. Under Japanese food labeling standards, nutrients present below threshold values may be labeled as 0 per 100 g or 100 mL, indicating that trace amounts may be present despite zero labeling. Moreover, because this study relied on label-derived nutritional data, the magnitude and direction of any deviation from the actual analyzed values remain unknown. Such discrepancies may have affected the classification of some products located near subcategory boundaries. Second, this study did not incorporate market data such as distribution volume, sales revenue, or market share. Therefore, the findings may not fully reflect the overall commercial landscape of sports foods in Japan. Although the use of market data could be considered, sports foods are widely consumed not only by athletes but also by the general population. As a result, sales and market share data inherently include the purchasing behavior of non-athlete consumers, making it difficult to distinguish athlete-specific usage patterns. Consequently, market-based indicators do not necessarily represent the actual usage among athletes. Given that this study focused on athlete use, product selection prioritized usage in sports settings rather than commercial distribution. Athletes are generally highly aware of anti-doping regulations and tend to exercise caution in supplement selection, often preferring products from trusted manufacturers to avoid inadvertent doping. Accordingly, this study selected products based on input from sports dietitians and focused on manufacturers with anti-doping certification, thereby reflecting real-world usage in athletic settings. Notably, companies added through expert consultation accounted for 28% of the total, and the final dataset included approximately 500 products, exceeding the scale of previous studies. These factors support the representativeness of the product list used in this study. Additionally, the exclusion of sports confectionery and liquid meals represents a limitation in the comprehensiveness of the exchange list. Although some sports confectionery products are used by athletes as supplementary snacks, the difficulty in establishing a consistent definition in the Japanese context precluded their inclusion. As definitions become more established and sport-specific products become more prevalent, future updates may consider incorporating these categories. Third, the exchange list has not been verified for practicality or effectiveness in real-world sports settings. Therefore, this study should be interpreted as a tool-development study aimed at constructing a sports food exchange list, rather than as a validation study evaluating its practical effectiveness. Future studies should evaluate its practicality among athletes and nutrition professionals. Finally, the data collection period was restricted to 27 March to 1 April 2025. As the sports nutrition market is subject to product turnover, including new product launches, discontinuations, and reformulations, the exchange list reflects the market at the time of data collection and requires periodic updates.

A booklet was also developed to facilitate practical implementation of the Japanese Sports Food Exchange List. It provides guidance not only for nutrition professionals in meal planning but also for athletes in applying the exchange list (Table S3). One intended application is to support athletes in independently selecting sports foods. The booklet is positioned as part of an applied framework to support the use of the exchange list in real-world settings, intended for use not only by certified sports dietitians and registered dietitians during meal planning, but also by athletes themselves. In practice, when dietitians develop meal plans, the booklet can facilitate efficient product selection based on target nutrient goals. For athletes, it enables autonomous and informed product selection even in situations where professional support is unavailable, such as during training camps or competitions. Future work should focus on implementing the exchange list and booklet in real-world sports settings and evaluating their practical utility and effectiveness. Use of the exchange list may also promote understanding of its underlying principles and encourage athletes to reflect on their sports food consumption practices.

## 5. Conclusions

This study developed a Japanese version of the Sports Food Exchange List, encompassing 498 products from 36 companies used by Japanese athletes. By classifying these products into 24 subcategories based on nutritional composition and defining representative values for each subcategory, the list enables product selection according to target nutrient requirements. Japanese sports foods exhibit distinct characteristics, including a wide distribution of nutritional composition, variability attributable to ingredient differences, and a high proportion of plant-based protein powders. Future work should focus on applying this exchange list in real-world sports settings and evaluating its effectiveness in supporting product selection. Regular updates are also required to reflect changes in the sports food market.

## Figures and Tables

**Table 1:** Final Target Distributors After Expert Selection.

	Target Distributors
1	Ai Robotics Inc. (Tokyo, Japan)
2	DNS (Tokyo, Japan)
3	GU Energy Labs (California, USA)
4	MYPROTEIN JAPAN K.K. (Tokyo, Japan)
5	Norm Co., Ltd. * (Kagoshima, Japan)
6	Optimum Nutrition (Illinois, USA)
7	ASAHI SOFT DRINKS CO., LTD. * (Tokyo, Japan)
8	Asahi Group Foods, Ltd. (Tokyo, Japan)
9	Ajinomoto Co., Inc. (Tokyo, Japan)
10	AEON TOPVALU CO., LTD. * (Chiba, Japan)
11	Ezaki Glico Co., Ltd. (Osaka, Japan)
12	Otsuka Pharmaceutical Co., Ltd. (Tokyo, Japan)
13	ALPRON Inc. (Tokyo, Japan)
14	ULTORA Inc. (Tokyo, Japan)
15	Meiji Co., Ltd. (Tokyo, Japan)
16	MAURTEN * (Gothenburg, Sweden)
17	Kirin Holdings Co., Ltd. (Tokyo, Japan)
18	SAURUS JAPAN Co., Ltd. (Osaka. Japan)
19	Suntory Holdings Limited * (Osaka. Japan)
20	CERRO TORRE JAPAN INC. (Chiba, Japan)
21	Taisho Pharmaceutical Co., Ltd. (Tokyo, Japan)
22	DAITO SUISAN CO., LTD. (Shizuoka, Japan)
23	DANONE JAPAN CO., LTD. * (Tokyo, Japan)
24	Coca-Cola (Japan) Company, Limited. * (Tokyo, Japan)
25	Nippon Shinyaku Co., Ltd. (Kyoto, Japan)
26	HOUSE WELLNESS FOODS CORPORATION (Hyogo, Japan)
27	Morinaga & Co., Ltd. (Tokyo, Japan)
28	Liv Laboratories Co., Ltd. (Tokyo, Japan)
29	BodyPlus International K.K. * (Miyagi, Japan)
30	VALX Inc. (Tokyo. Japan)
31	Aristo Co., Ltd. * (Tokyo. Japan)
32	NatureLab. Co., Ltd. (Tokyo, Japan)
33	Peak Performance Nutrition (Tokyo, Japan)
34	Real Style Co., Ltd. (Nara, Japan)
35	Amway Japan G.K. (Tokyo, Japan)
36	Hamari Nutritional Sciences (Osaka, Japan)

* Added by the distributor based on advice from a registered dietitian and expert selection.

**Table 2:** Selection Criteria and Method for Setting One Unit for Target Products in 7 Categories in the Japanese Sports Food Exchange Table.

Categories	Selection Criteria *	1 Unit Setting
Sports drinks	Products sold in liquid form	One bottle is defined as one unit. When multiple products with the same nutritional composition but different contents exist, a 500 mL bottle shall be considered as one unit.
Energy jellies	Products sold in a jelly-like form using spout containers	One spout container is defined as one unit.
Energy bars	Products sold in bar form	The serving size indicated by the manufacturer is defined as one unit. For products in which the serving size was not specified, one bag is considered as one unit.
Energy gels	Products sold in viscous or thickened forms	The serving size indicated by the manufacturer is defined as one unit. For products in which the serving size was not specified, one bag is considered as one unit.
Protein drinks	Products sold in liquid form	One bottle is defined as one unit.
Protein bars	Products sold in bar form	The serving size indicated by the manufacturer is defined as one unit. For products in which the serving size was not specified, one bag is considered as one unit.
Protein powders	A powdered product that is dissolved in water or milk and consumed.	The single serving size indicated by the manufacturer was defined as one unit.

* Applies to products that can be stored at room temperature across all 7 categories.

**Table 3:** Acceptable Standards for Energy and Major Nutrient Distribution within Each Subcategory of the Japanese Sports Food Exchange Table *.

	Standard Deviation (SD) for Each Group	Coefficient of Variation for Each Group (CV)	z Values for Each Food
Energy	20 kcal	30%	±2
Protein	3 g		
Fat	2 g		
Carbohydrate	5 g		

* This study adopted the criteria reported by Wheeler et al. [30].

**Table 4:** List of Excluded Items in the Subcategory Classification of the Japanese Sports Food Exchange Table.

Sports drinks		
z	>2	Kirin Holdings Co., Ltd.: Salty Lychee †
Non-targeted product intake		Coca-Cola (Japan) Company, Limited: aquarius NEWATER
		Coca-Cola (Japan) Company, Limited: aquarius ZERO
		Meiji Co., Ltd.: VAAM SMART FIT WATER Lemon Flavor
		Meiji Co., Ltd.: VAAM SMART FIT WATER Apple Flavor
		SAURUS JAPAN Co., Ltd.: SAURUS Sports Drink Light
Energy jellies		
z	>2	Morinaga & Co., Ltd.: in Jelly Protein 15 g Mixed Berry Yogurt Flavor ‡
		DNS: Protein Jel-X Meal Jelly, Tropical Fruit Flavor ‡
z	<−2	Meiji Co., Ltd.: Instant Energy Jelly Amino Acids & Royal Jelly Zero Sugar—Nutritional Drink Flavor †
		Meiji Co., Ltd.: VAAM Smart Fit Jelly †
		Morinaga & Co., Ltd.: in Jelly Protein Yogurt Flavor †
Energy gels		
z	>2	Taisho Pharmaceutical Co., Ltd.: Lipovitan Jelly for Emergency Storage †
z	<−2	DAITO SUISAN Co., Ltd.: OREHA SESSHUSU RECOVERY Yuzu Flavor †
Protein bars		
z	<−2	Morinaga & Co., Ltd.: in-bar protein granola §
Protein powders		
z	>2	MYPROTEIN JAPAN K.K.: Impact Diet Whey—Chocolate Smooth ‡
		Optimum Nutrition: Serious Mass, Chocolate ‡
		Optimum Nutrition: Serious Mass, Vanilla ‡
		Optimum Nutrition: Serious Mass, Kulfi ‡
z	<−2	Norm Co., Ltd.: NORM Pro Energy Charge ‡
		Meiji Co., Ltd.: SAVAS PRO WPI Recovery Muscat Flavor ‡
		ALPRON Inc.: Weight Up Protein ‡
		Morinaga & Co., Ltd.: Recovery Power Protein Cocoa Flavor ‡
		Morinaga & Co., Ltd.: Recovery Power Protein Peach Flavor ‡
		Ezaki Glico Co., Ltd.: EXTRA AMINO ACID PROTEIN Sour Milk Flavor ‡
		Amway Japan G.K.: Nutrilite All Plant Protein ‡

† Carbohydrate, ‡ Protein, § Fat.

**Table 5:** Major Nutrient Content per Unit for Each of the 7 Categories in the Japanese Sports Food Exchange Table (Total of 498 Products).

	*n*	1 Unit of Mass (g)	Energy (kcal)					Protein (g)					Fat (g)					Carbohydrate (g)				
			Mean	SD	Min	Max	CV	Mean	SD	Min	Max	CV	Mean	SD	Min	Max	CV	Mean	SD	Min	Max	CV
Sports drinks	17	513	84	20	53	125	0.2	1	1	0	5	2.4	0	0	0	0	0	1	0	0	1	0.2
Energy jellies	37	181	140	32	100	250	0.2	2	48	55	200	21.5	0	3	0	10	8.7	0	11	13	50	48.3
Energy bars	34	33	156	6	22	40	0	3	33	96	200	10.5	8	2	1	7	0.2	19	3	3	11	0.1
Energy gels	44	40	110	14	94	157	0.1	1	2	0	8	2.8	0	1	0	9	4.1	0	0	0	1	0.8
Protein drinks	24	250	113	23	70	151	0.2	19	5	10	29	0.3	1	2	0	5	1.6	7	4	1	13	0.7
Protein bars	39	47	216	40	137	332	0.2	18	4	10	30	0.2	10	3	3	17	0.3	14	3	8	21	0.2
Protein powders	303	32	118	57	51	483	0.5	21	5	8	50	0.2	1	1	0	7	0.9	6	10	0	68	1.6

Data was collected in milliliters for sports drinks and protein drinks, and in grams for other products. SD: standard deviation, Min: minimum value, Max: maximum value, CV: Coefficient of Variation.

**Table 6:** Nutritional Composition per Unit for All 24 Subcategories in the Japanese Sports Food Exchange Table.

	*n*	1 Unit of Mass (g)	Energy (kcal)			Protein (g)			Fat (g)			Carbohydrate (g)			Salt Equivalent (g)		
			Mean	SD	CV	Mean	SD	CV	Mean	SD	CV	Mean	SD	CV	Mean	SD	CV
Sports drinks (SD)																	
SD1	9	536	70	11	0.2	1	2	1.6	0	0	0	16	2	0.1	0.6	0.1	0.2
SD2	8	488	99	16	0.2	0	0	0	0	0	0	24	4	0.2	0.6	0.1	0.2
Energy jellies (EJ)																	
EJ1	20	168	98	16	0.2	2	3	2	0	0	0	24	5	0.2	0.2	0.2	0.9
EJ2	13	192	188	13	0.1	1	2	1.1	0	0	0	46	4	0.1	0.2	0.1	0.6
EJ3	4	206	193	13	0.1	9	1	0.1	4	2	0.4	33	1	0	0.1	0	0.4
Energy bars (EB)																	
EB1	16	28	127	18	0.1	4	2	0.5	7	2	0.3	13	2	0.2	0.2	0.1	0.4
EB2	15	38	187	14	0.1	3	1	0.5	10	2	0.2	23	2	0.1	0.2	0.1	0.6
EB3	3	40	150	5	0	2	0	0.1	3	0	0.1	28	1	0	0.4	0.5	1.3
Energy gels (EG)																	
EG1	22	37	100	3	0	0	0	0	0	1	2.3	23	1	0	0.2	0.1	0.3
EG2	17	42	115	6	0.1	0	0	2.2	0	0	4	29	2	0.1	0.1	0.1	1.1
EG3	5	48	138	16	0.1	7	2	0.2	2	4	2	28	3	0.1	0.5	0.2	0.4
Protein drinks (PD)																	
PD1	15	233	101	17	0.2	16	3	0.2	0	0	2.6	9	3	0.4	0.3	0.3	0.8
PD2	6	243	139	14	0.1	22	2	0.1	4	0	0.1	3	2	0.8	0.4	0.3	0.7
PD3	3	350	123	1	0	29	0	0	0	0	0	1	0	0.2	0	0	0
Protein bars (PB)																	
PB1	16	41	190	21	0.1	15	2	0.1	10	3	0.3	13	1	0.1	0.3	0.1	0.4
PB2	12	52	207	11	0.1	19	2	0.1	8	1	0.2	16	4	0.2	0.3	0.1	0.3
PB3	8	48	242	12	0.1	19	3	0.2	14	1	0.1	11	2	0.2	0.7	0.3	0.4
PB4	3	63	321	11	0	28	3	0.1	17	0.4	0	16	0	0	0.4	0.3	0.3
Protein powders (PP)																	
PP1	47	22	75	13	0.2	13	3	0.2	1	1	0.7	4	4	0.8	0.4	0.2	0.6
PP2	20	37	131	18	0.1	18	3	0.2	1	1	0.8	15	4	0.3	0.3	0.2	0.5
PP3	224	30	113	12	0.1	22	2	0.1	1	1	0.6	4	2	0.5	0.3	0.2	0.8
PP4	4	125	456	38	0.1	40	6	0.1	7	0	0	63	7	0.1	0.1	0.1	1
PP5	4	100	379	7	0	30	1	0	6	0	0.1	51	1	0	0.2	0.1	0.7
PP6	4	68	273	25	0.1	22	2	0.1	4	2	0.6	39	4	0.1	0.2	0.1	0.6

Data was collected in milliliters for sports drinks and protein drinks, and in grams for other products. Salt equivalent (g) is calculated by multiplying sodium (mg) by 2.54/1000. SD: standard deviation, CV: Coefficient of Variation.

**Table 7:** Cross-category exchangeable pairs for carbohydrate and protein supplementation in the Japanese Sports Food Exchange Table.

	Subcategory	Number of Units	1 Unit of Mass (g)	Average Value for Each Subcategory			
				Energy (kcal)	Protein (g)	Fat (g)	Carbohydrate (g)
Sports Food for Carbohydrate Replenishment							
1	EJ1	1 Unit	168	98	1.5	0	24
	EG1	1 Unit	37	100	0	0	23
2	SD2	1 Unit	488	99	0	0	24
	EJ1	1 Unit	168	98	1.5	0	24
3	SD2	1 Unit	488	99	0	0	24
	EG1	1 Unit	37	100	0	0	23
4	SD2	2 Unit	488	198	0	0	48
	EJ2	1 Unit	192	188	1.4	0	46
5	SD1	2 Unit	536	140	2	0	32
	EB3	1 Unit	40	150	2	3	28
Sports Food for Protein Supplementation							
6	PP3	1 Unit	30	113	22	1	4
	PD1	1 Unit	233	101	16	0	9

EJ: Energy jellies, EG: Energy gels, SD: Sports drinks, EB: Energy bars, PP: Protein powders, PD: Protein drinks; Data was collected in milliliters for sports drinks and protein drinks, and in grams for other products; When exchanging sports drinks across categories, note that hydration and electrolyte levels are not taken into account.

**Table 8:** System Usability Scale scores.

	1	2	3	4	5	6	7	8	9	10	SUS Score
Respondent 1	3	1	2	4	4	3	1	4	2	4	40
Respondent 2	4	3	4	5	2	3	2	3	2	5	38
Respondent 3	5	3	4	4	4	2	3	3	3	4	58
Respondent 4	4	2	4	3	4	2	4	2	3	5	63
Respondent 5	3	2	4	2	4	2	3	2	4	2	70
Respondent 6	3	3	4	4	3	3	2	3	3	2	50
Mean											53

## Data Availability

The original data presented in this study are included in the article and Supplementary Materials. Further inquiries can be directed to the corresponding author.

## Supplementary Materials

The following supporting information can be downloaded at https://www.mdpi.com/article/10.3390/nu18111711/s1. Table S1: Companies Excluded After Expert Selection by 7 Category, Table S2: Target Product List, Table S3: Brochure on How to Use.

## Abbreviations

IOC	International Olympic Committee
AIS	supplement classification of the Australian Institute of Sport
FSANZ	food standards of the Australia New Zealand Food Standards Code
JSPO	Japan Sport Association
JADA	Japan Anti-Doping Agency
SD	sports drinks
EJ	energy jellies
EB	energy bars
EG	energy gels
PD	protein drinks
PB	protein bars
PP	protein powders
SD	Standard Deviation
Min	minimum value
Max	maximum value
CV	Coefficient of Variation
