# Supplementary material for: Development of a Japanese Sports Food Exchange List Reflecting Products Used in Japanese Athletic Settings
Source: Nutrients. 2026 May 27;18(11):1711. doi: 10.3390/nu18111711 (PMC13259277; doi:10.3390/nu18111711)
Supplement: Supplementary file 1 [file nutrients-18-01711-s001.zip › Table S3.pdf]

Table S3: Brochure on How to Use

1 What Are Sports Foods?

Athletes require large amounts of energy and nutrients on a daily basis due to high-intensity physical activity and prolonged training sessions. While a food-first approach is recommended for athletes' nutritional management, the use of sports foods and supplements can be effective in certain situations.[1)2)3)]。

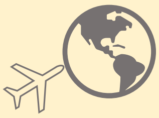

Away games

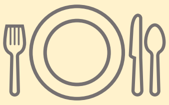

When you're on a special diet

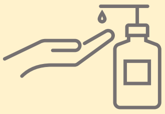

When you have concerns about food safety

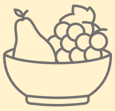

When you are deficient in certain nutrients

Sports Food

Sports food refers to foods or beverages designed to provide athletes with a quick and convenient source of energy and nutrients, particularly before and after exercise[4) 5) 6)]。

Sports Food

Foods and beverages designed to provide athletes with a quick and convenient way to replenish energy and nutrients during nutritional support

Examples: sports drinks, protein powders, etc.

Medical Supplements

Products prescribed to prevent or treat clinical conditions, such as when a nutritional deficiency is diagnosed

Examples: Iron, calcium, etc.

Ergogenic aids

Products designed to enhance performance

Examples: caffeine, creatine, etc.

2 What is a Sports Food Exchange List?

This table categorizes sports foods commonly used in athletic settings based on their nutritional characteristics. It serves as a useful tool for developing meal plans for athletes.

The table covers 7 types of sports foods. Since these products can be stored at room temperature, they are convenient to carry.

▶Sports foods used for carbohydrate replenishment

①Sports drinks (SD): Products sold in liquid form

②Energy jellies (EJ): Products sold in jelly form, often in spouted containers

③Energy bars (EB): Products sold in bar form

④Energy gels (EG): Products sold in a thick, viscous gel form

▶Sports foods used for protein supplementation

⑤Protein drinks (PD): Products sold in liquid form

⑥Protein bars (PB): Products sold in bar form

⑦Protein powder (PP): Products sold in powder form that are mixed with water and consumed

About 1 Unit

Regardless of the form or type of product, 1 unit has been established as a standard for comparing and organizing energy content and the amounts of protein, fat, and carbohydrates.

- Sports drinks: One bottle is defined as one unit. When multiple products with the same nutritional composition but different contents exist, a 500 ml bottle shall be considered as one unit.
- Energy jellies: One spout container is defined as one unit.
- Energy bars, Energy gels, Protein bars
  ： The serving size indicated by the manufacturer is defined as one unit. For products in which the serving size was not specified, one bag is considered as one unit.
- Protein drinks： One bottle is defined as one unit.
- Protein powders： One unit is defined as the single serving size indicated by the manufacturer.

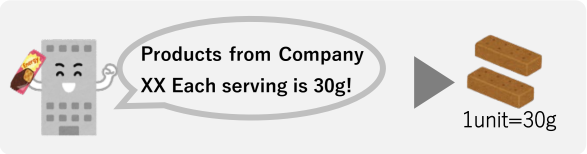

3 About the Target Categories

The nutritional composition per unit and product examples for all 24 subcategories are shown below.
(E: Energy, P: Protein, F: Fat, C: Carbohydrates)

Sports Food for Carbohydrate Replenishment

Sports drinks

SD1

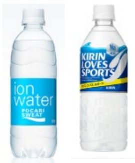

E 70kcal
P 1g
F 0g
C16g

SD2

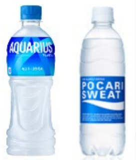

E 99kcal
P 0g
F 0g
C24g

Energy jellies

EJ1

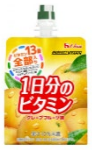

E 98kcal
P 2g
F 0g
C 24g

EJ2

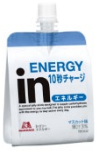

E 188kcal
P 1g
F 0g
C 46g

EJ3

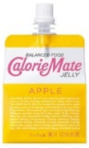

E 193kcal
P 9g
F 4g
C 33g

Energy bars

EB1

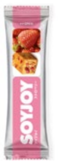

E 127kcal
P 4g
F 7g
C 13g

EB2

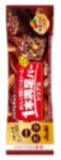

E 187kcal
P 2.5g
F 10g
C 23g

EB3

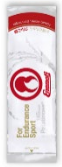

E 150kcal
P 2g
F 3g
C 28g

Energy gels

EG1

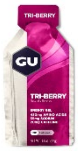

E 100kcal
P 0g
F 0g
C 23g

EG2

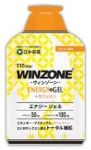

E 115kcal
P 0g
F 0g
C 29g

EG3

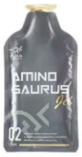

E 138kcal
P 7g
F 2g
C 28g

Sports Food for Protein Replenishment

Protein drinks

PD1

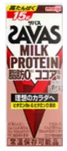

E 101kcal
P 16g
F 0g
C 9g

PD2

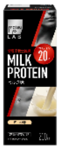

E 139kcal
P 22g
F 4g
C 3g

PD3

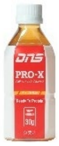

E 123kcal
P 29g
F 0g
C 1g

Protein bars

PB1

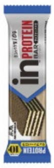

E 195kcal
P 15g
F 10g
C 13g

PB2

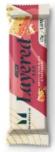

E 207kcal
P 19g
F 8g
C 16g

PB3

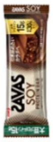

E 242kcal
P 19g
F 14g
C 11g

PB4

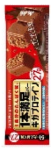

E 321kcal
P 28g
F 17g
C 16g

Protein powders

PP1

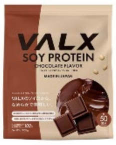

E 75kcal
P 13g
F 1g
C 4g

PP2

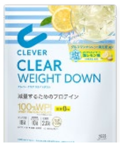

E 131kcal
P 18g
F 1g
C 15g

PP3

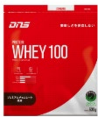

E 113kcal
P 22g
F 1g
C 4g

PP4

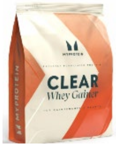

E 456kcal
P 40g
F 1g
C 4g

PP5

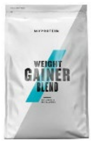

E 379kcal
P 30g
F 6g
C 51g

PP6

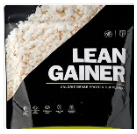

E 273kcal
P 22g
F 4g
C 39g

4 How to Exchange Items

★Exchanging within the same subcategory

You can exchange items on a per-unit basis within Subcategory 1 of Energy Jelly. You cannot exchange items with Subcategories 2 or 3 of Energy Jelly.

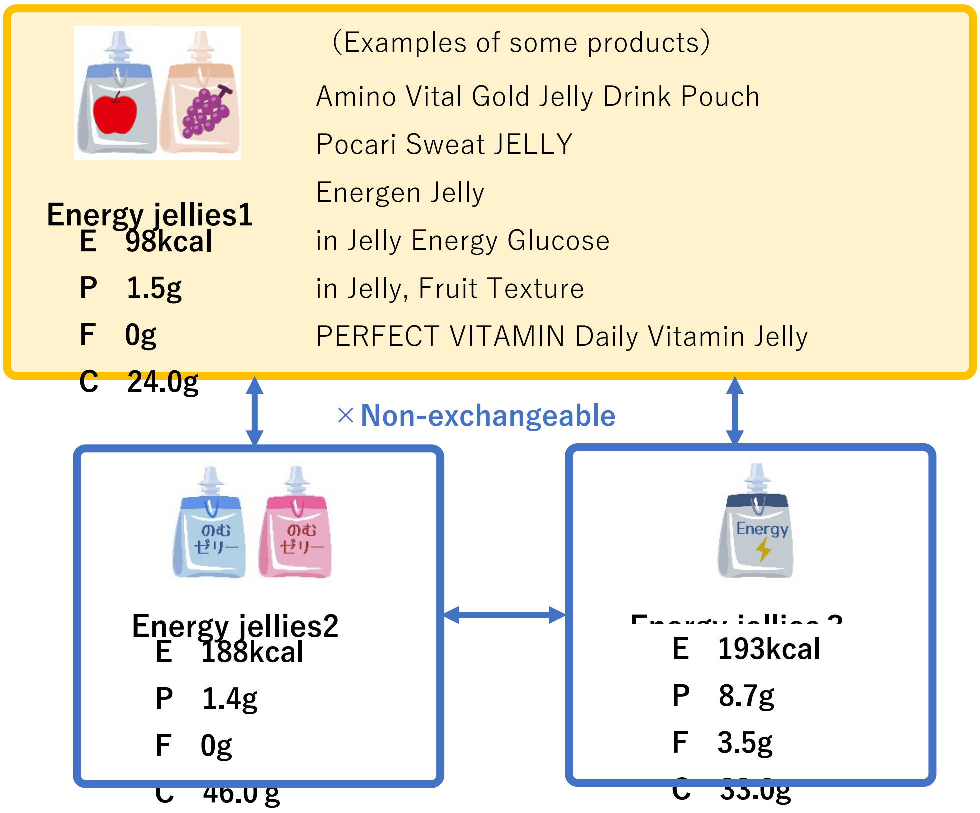

★Cross-Category Exchanges

Cross-category exchanges are available in certain subcategories. The exchangeable pairs are listed below.

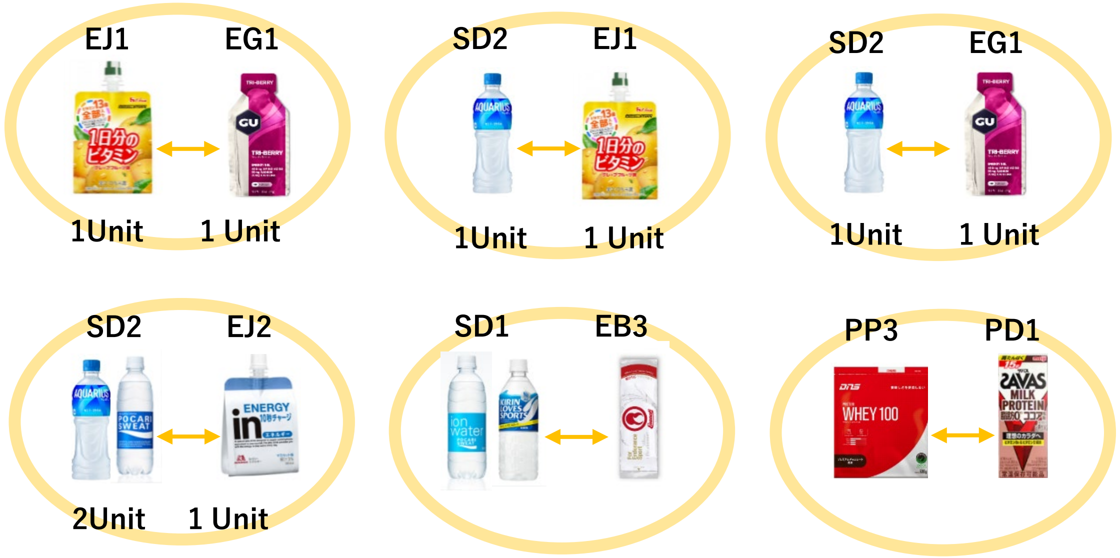

2Unit	1 Unit	1Unit	1 Unit

5 Points to Note When Using the Exchange List

○Food-First Meal Planning

While sports foods and supplements offer a convenient way to replenish nutrients, they should not be consumed indiscriminately. It is important to establish a solid foundation of regular meals and then select these products appropriately only when necessary, such as during away games or periods of high physical activity. When using sports foods, carefully consider whether your regular diet is insufficient and what specific benefits these products offer, and make choices that align with your goals and circumstances.

Has its effectiveness been verified?

Is there any risk of foreign substances being mixed in that could result in a doping violation?

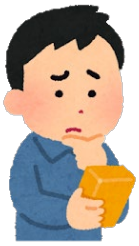

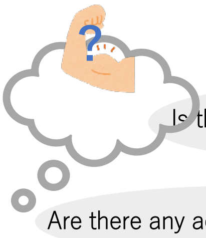

Is the price reasonable?

Are there any adverse health effects?

Examples of foods high in carbohydrates

Carbohydrates 15g
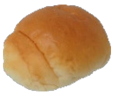
Rolled bread 1
(30g)

Carbohydrates 37g
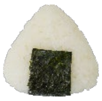
Rice ball 1
(100g)

Carbohydrates 40g
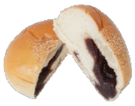
Red bean bun 1
(80g)

Carbohydrates 23g
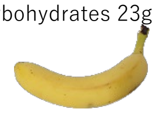
Banana 1
(100g)

Carbohydrates 54g
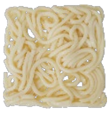
Udon
(250g)

Carbohydrates 73g
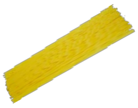
Pasta
(100g)

Examples of foods high in proteins

Protein 3g
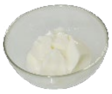
Yogurt
(90g)

Protein 3g
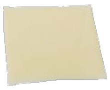
Cheese 1
(15g)

Protein 6g
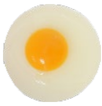
Eggs
(50g)

Protein 7g
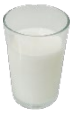
Milk
(200g)

Protein 7g
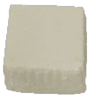
Tofu
(100g)

Protein 12g
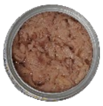
Canned tuna
(70g)

Protein 15g
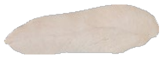
Chichen breast
(50g)

Protein 23g
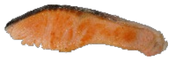
Salmon
(80g)

〇Choosing Sports Foods Based on Consumption Situations

Even among sports foods with the same nutritional composition, there are products that come in different forms. We recommend selecting sports foods based not only on their nutritional composition but also on their form, depending on the specific consumption situation.

Example)

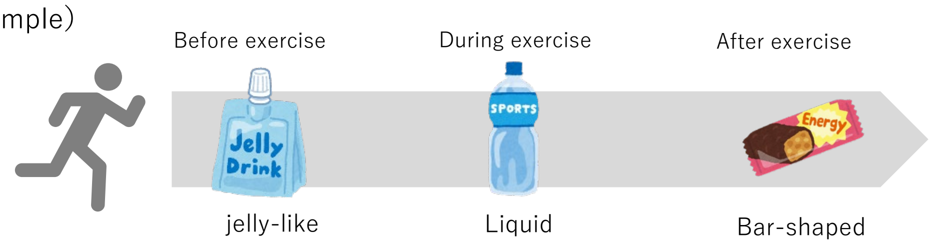

〇Use of Sports Drinks

Sports drinks are used during prolonged exercise in hot and humid conditions not only to replenish carbohydrates but also to replenish fluids and electrolytes. Therefore, if your goal is to replenish sodium and fluids, you should exercise caution when substituting sports drinks with sports foods intended solely for carbohydrate replenishment, as this may make it difficult to achieve your primary goal of replenishing fluids and sodium.

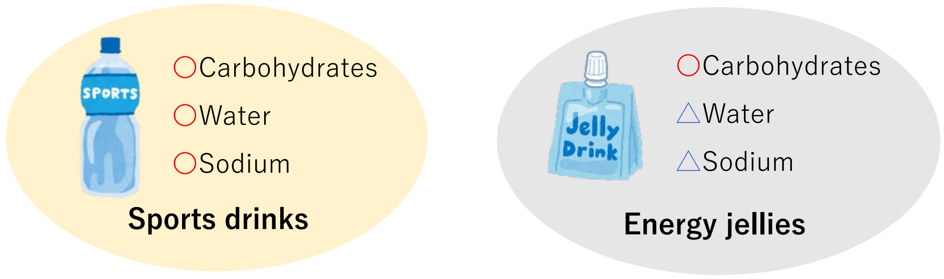

〇Note the unit size

Even within the same product category, the unit size (the manufacturer's recommended serving size) may vary significantly. Please check the product list or the manufacturer's product information for details.

Example)

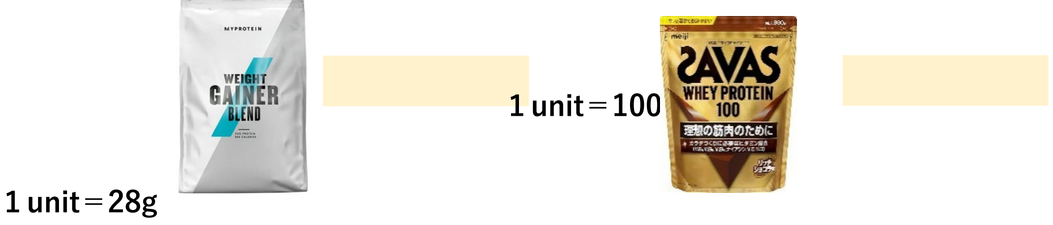

MYPROTEIN
Weight Gainer Blend

SAVAS
Whey Protein 100

6 How to use it

①How to use sports foods in combination with other sports foods depending on the competition situation

Assuming a male weighing 65kg running a full marathon

	Target nutrient intake [7]	Examples of proposals utilizing exchange list
Before the race	Consume 1-4g/kg of carbohydrates.<br>(Reduce fat and protein intake according to your gut health.)	SD2 × 1 unit/h<br>EJ2 × 1 unit/h<br>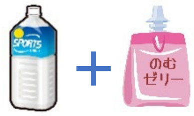<br>By selecting one product from SD2 and one product from EJ2, you can consume approximately 70g of carbohydrates in total.
～1 hour from start	Small amount of carbohydrates	SD 1 × 1 unit/h<br>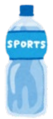<br>If you select one product from SD1, you can consume approximately 16g of carbohydrates.
1 to 2.5 hours	30～60g/h	SD2 × 1 unit/h<br>EG1 × 1 unit/h<br>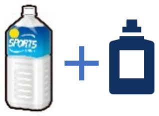<br>By selecting one product from SD2 and one product from EG1, you can consume approximately 47g of carbohydrates in total.
2． 5 hours～	Max 90g/h	SD2 × 1 unit/h<br>EG2 × 1 unit/h<br>EB2 × 1 unit/h<br>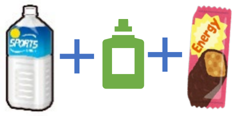<br>By selecting one product from SD2, one from EG2, and one from EB2, you can consume approximately 76g of carbohydrates in total.

②When proposing product selection

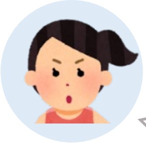

athlete

I want to drink a protein powder that allows me to get protein while keeping fat intake low.

So, how about choosing a product from subcategory 3 (PP3) of protein powder? In this subcategory, one unit provides approximately 22g of protein and approximately 1g of fat.

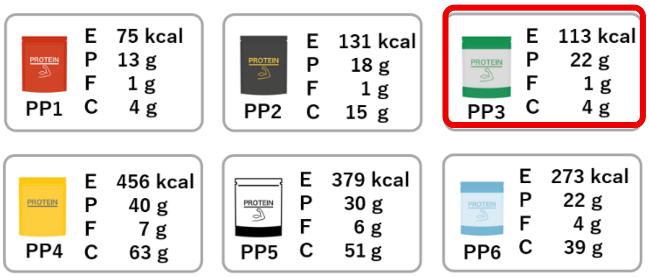

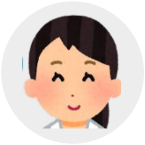

Registered Dietitian

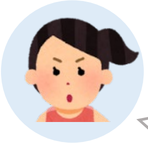

I see! Hmm, I'm torn between product 〇〇 and product △△ in subcategory 3 (PP3). Which one should I choose...?

When choosing a product, it's important to consider the risk of doping. Between these two, 〇〇 has received the Informed Choice rating.

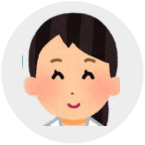

▶プロテインパウダー（PP）

			販売元	ブランド	商品名	フレーバー	1単位の質量（g）	エネルギー（kcal）	たんぱく質（g）	脂質（g）	炭水化物（g）
PP3		1	A社	Aブランド	・・・	プレーン	**30**	116	24	1.6	4.6
	★	2	A社	Aブランド	〇〇	チョコレート	**35**	134	22	1.7	8.7
		3	B社	Bブランド	・・・	抹茶	**25**	91	21	0.375	2.55
		4	C社	Cブランド	△△	チョコレート	**30**	108	21.7	1.05	3
	☆	5	C社	Cブランド	・・・	ヨーグルト	**30**	114	23.3	0.8	3.5

★INFORMEDCHOICE獲得商品（2025/08/01）
☆アンチドーピングのためのスポーツサプリメント製品情報公開サイト掲載商品（2025/08/01）

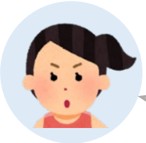

Okay, then I'll try the protein powder from〇〇 !

7　Examples of eligible products

Sports drinks category (SD)

Sports drinks　subcategory 1

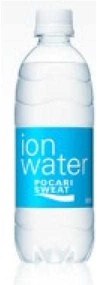

Otsuka Pharmaceutical Co., Ltd.

POCARI SWEAT ION WATER

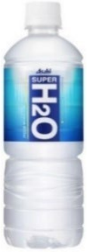

Asahi Soft Drinks Co., Ltd.

Asahi Super H2O

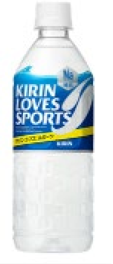

Kirin Holdings Co., Ltd.

KIRIN LOVES SPORTS

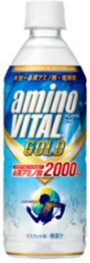

Kirin Holdings Co., Ltd.

Amino Vital Gold 2000 Drink

Sports drinks　subcategory 2

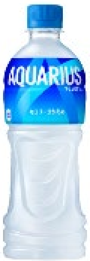

Coca-Cola Company, Limited.

Aquarius

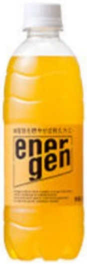

Otsuka Pharmaceutical Co., Ltd.

Energen

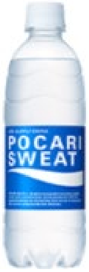

Otsuka Pharmaceutical Co., Ltd.

POCARI SWEAT

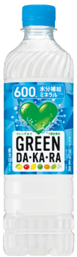

Suntory Holdings Limited

GREEN　DA・KA・RA

Energy jellies　category (EJ)

Energy jellies　subcategory 1

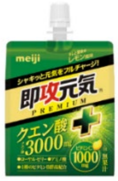

Meiji Co., Ltd.

Sokko Genki Jelly Amino Acid & Royal Jelly

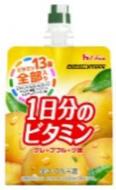

HOUSE　WELLNESS　FOODS CORPORATION

PERFECT VITAMIN

Daily Vitamin Jelly

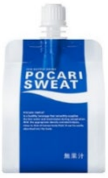

Otsuka Pharmaceutical Co., Ltd.

Pocari Sweat JELLY

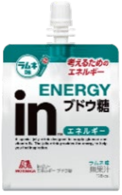

Morinaga & Co., Ltd.

in Jelly Energy Glucose

Energy jellies　subcategory 2

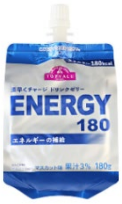

AEON TOPVALU CO., LTD.

Quick Charge Drink Jelly

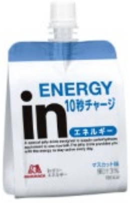

Morinaga & Co., Ltd.

in Jelly Energy

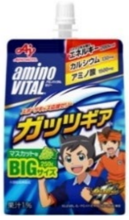

Ajinomoto Co., Inc.

amino Vital Jelly Drink Guts Gear

Energy jellies　subcategory 3

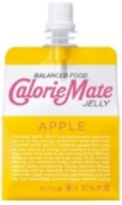

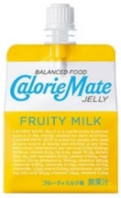

Otsuka Pharmaceutical Co., Ltd.

Calorie Mate JELLY

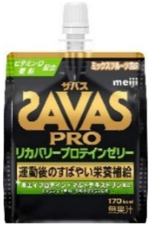

Meiji Co., Ltd.

SAVAS PRO Recovery Protein Jelly

Energy bars　category (EB)

Energy bars　subcategory 1

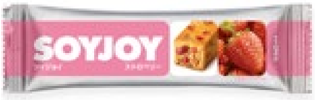

Otsuka Pharmaceutical Co., Ltd.

SOYJOY

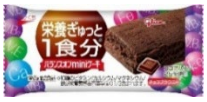

Ezaki Glico Co., Ltd.

Balance On Mini Cake

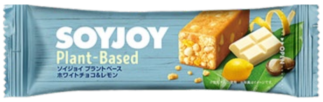

Otsuka Pharmaceutical Co., Ltd.

SOYJOY　Plant-Based

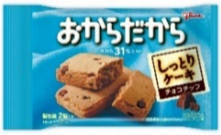

Ezaki Glico Co., Ltd.

Okara Dakara

Energy bars　subcategory 2

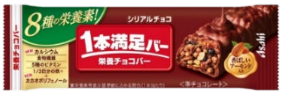

Asahi Group Foods, Ltd.

Ippon Manzoku Bar

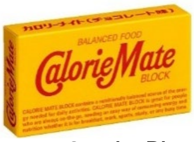

Otsuka Pharmaceutical Co., Ltd.

SOYJOY

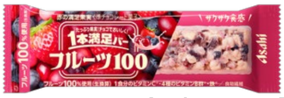

Asahi Group Foods, Ltd.

Ippon Manzoku Bar

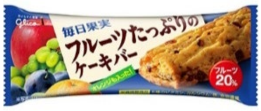

Ezaki Glico Co., Ltd.

Fruit Cake Bar

Energy bars　subcategory 3

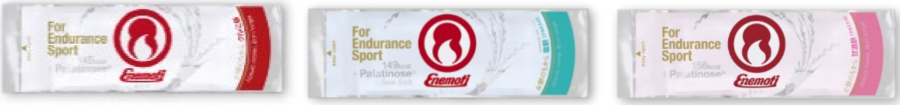

CERRO TORRE JAPAN INC.

Enemoti

Energy gels　category (EG)

Energy gels　subcategory 1

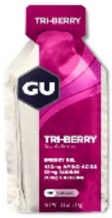

GU Energy Labs

ORIGINAL ENEGY GEL

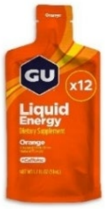

GU Energy Labs

Liquid Energy Gel

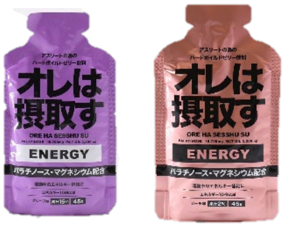

DAITO SUISAN CO.,LTD.

Ore wa Sesshus ENERGY

Energy gels　subcategory 2

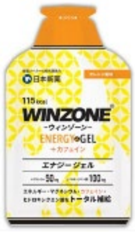

Nippon Shinyaku Co., Ltd.

WIZONE

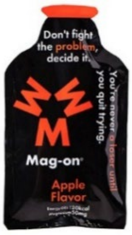

CERRO TORRE JAPAN INC.

Mag-O n　Energy Gel

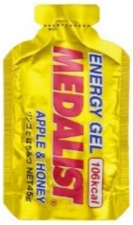

Aristo Co., Ltd.

MEDALIST ENERGY GEL

Energy gels　subcategory 3

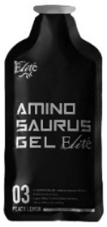

SAURUS JAPAN Co., Ltd.

AMINO SAURUS gel Elite

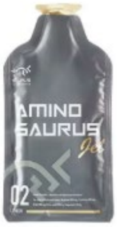

SAURUS JAPAN Co., Ltd. AMINO

SAURUS gel

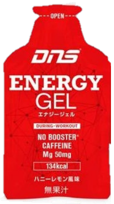

DNS

ENERGY GEL

Protein drinks　category (PD)

Protein drinks subcategory 1

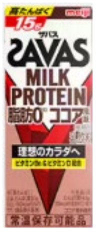

Meiji Co., Ltd.

SAVAS

MILK PROTEIN 0% Fat

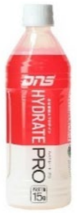

DNS

HYDRATE PRO

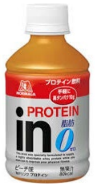

Morinaga & Co., Ltd.

in PROTEIN　PET

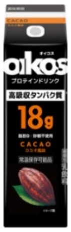

DANONE JAPAN CO., LTD.

oikos

Protein Drink, High Absorption Protein 18g

Protein drinks subcategory 2

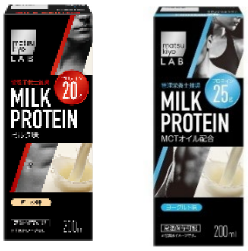

Liv Laboratories Co., Ltd.

matsukiyo LAB

Milk　Protein

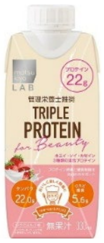

Liv Laboratories Co., Ltd.

matsukiyo LAB

TRIPLE PROTEIN DRINK

Protein drinks subcategory 3

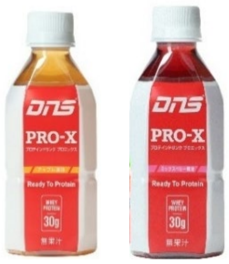

DNS

Pro-X

Protein bars　category (PB)

Protein bars subcategory 1

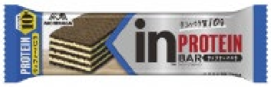

Morinaga & Co., Ltd.

in BAR Protein

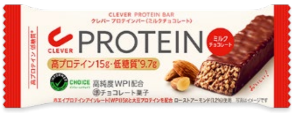

NatureLab. Co., Ltd.

CLEVER MUSCLE

Protein Bar

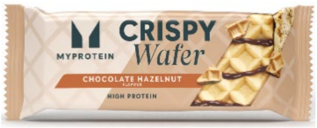

MYPROTEIN

Protein Wafer

Protein bars subcategory 2

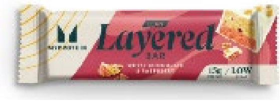

MYPROTEIN

Layered Protein Bar

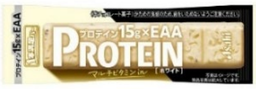

Asahi Group Foods, Ltd.

Ippon Manzoku Bar

Protein series

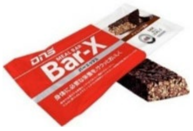

DNS

MEAL BAR Bar-X

Protein bars subcategory 3

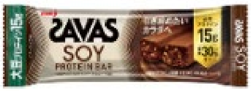

Meiji Co., Ltd.

SAVAS

Soy Protein Bar

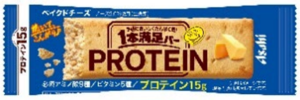

Asahi Group Foods, Ltd.

Ippon Manzoku Bar

Protein series

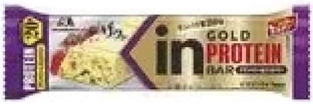

Morinaga & Co., Ltd.

in Bar Protein GOLD

Protein bars subcategory 4

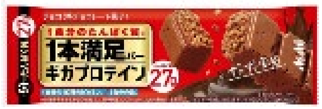

Morinaga & Co., Ltd.

Giga Protein Series

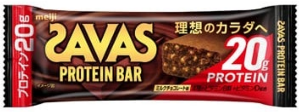

Meiji Co., Ltd.

SAVAS

Protein Bar

Protein powders　category (PP)

Protein powders　subcategory 1

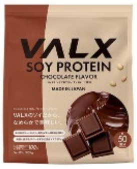

VALX Inc.

Soy Protein

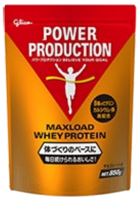

Ezaki Glico Co., Ltd.

POWER PRODUCTION

Max Load Whey Protein

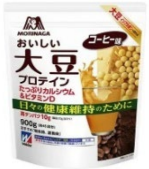

Morinaga & Co., Ltd.

Delicious Soy Protein

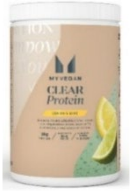

MYPROTEIN

Clear Vegan Protein Powder

Protein powders　subcategory 2

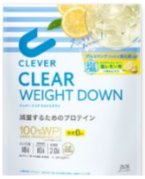

NatureLab.　Co.,　Ltd.

CLEVER MUSCLE

Weight Down Protein

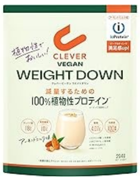

NatureLab.　Co.,　Ltd.　CLEVER MUSCLE

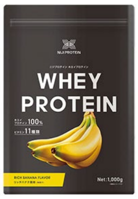

AiROBOTICS

NIJI PROTEIN

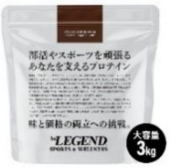

Real Style Co.,Ltd.

Be LEGEND Sports & Wellness

Protein powders　subcategory 3

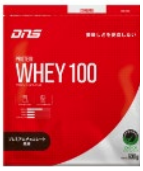

DNS

Protein Whey 100

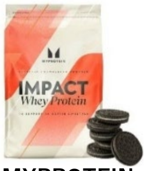

MYPROTEIN

Impact Whey Protein

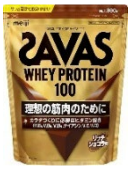

Meiji Co., Ltd.

SAVAS

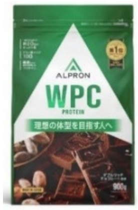

ALPRON Inc.

Whey Protein WPC

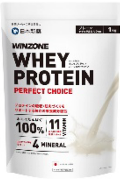

Nippon Shinyaku Co., Ltd.

WINZONE

Whey Protein

Protein powders　subcategory　4

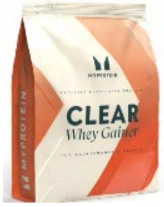

MYPROTEIN

Clear Whey Mass Gainer

Protein powders　subcategory 5

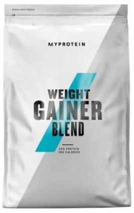

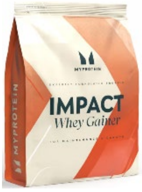

(Different packaging)

MYPROTEIN

Impact Weight Gainer

Protein powders　subcategory 6

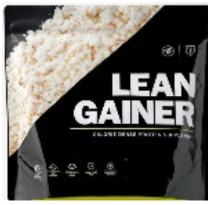

BodyPlus International K.K.

HALEO LEAN GAINER

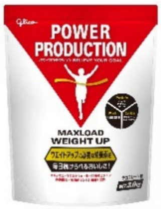

Ezaki Glico Co., Ltd.

POWER PRODUCTION

MAXLOAD WEIGHT UP

7 References

1）Yasuda J, Myoenzono K, Takai E, et al., Importance of "meal first" strategy and effective situations of supplement use in elite athletes: Japan high performance sport center position stand, *Front Sports Act Living*, 5, 1188224 (2023)

2）Collins J, Maughan RJ, Gleeson M, et al., UEFA expert group statement on nutrition in elite football, Current evidence to inform practical recommendations and guide future research. *Br J Sports Med*, 55, 416 (2021)

3）I. Garthe, R. Ramsbottom, Elite athletes, a rationale for the use of dietary supplements: A practical approach, *PharmaNutrition*, 14, 10, 100234, (2020)

4) Maughan RJ, Burke LM, Dvorak J, et al., IOC consensus statement: dietary supplements and the high-performance athlete, *Br J Sports Med*, 52, 439-455, (2018)

5)The Australian Institute of Sport,https://www.ausport.gov.au/ais/nutrition/supplements,（accessed on 5 December 2025）

6)Japan Sport Association. 2024 Consensus on Supplement Use and Application—Athlete Edition. Available online: https://www.japan-sports.or.jp/Portals/0/data/supoken/doc/supplement/consensus/2024/supplement_consensus_2024_athlete_2p.pdf. (accessed on 1 April 2025).

7) Burke LM, Jeukendrup AE, Jones AM, Mooses M. Contemporary Nutrition Strategies to Optimize Performance in Distance Runners and Race Walkers. *Int J Sport Nutr Exerc Metab*. 29, 2, 117-129, (2019)
